# Transactions Medical and Chirurgical Faculty of Maryland

**Published:** 1874-09

**Authors:** 


Transactions of the Medical and Chirurgical Faculty of the State of Maryland, seventy-fifth annual session, held at Baltimore, Maryland, April, 1874.

Appendix: Annual oration, by Lewis H. Steiner, M.D.; report of section on Surgery, by Christopher Johnston, M.D., (illustrated;) report of section on Practice and Obstetrics, by John Morris, M.D.; report of the section on Materia Medica, Pharmacy, etc., by S. C. Chew, M.D.; report of the section on Meterology, Epidemics, etc., by Hiram J. Penrod, M.D., (illustrated;) report of section on Anatomy, Physiology, etc., by F. T. Miles, M.D.; report of section on Psycology and Medical Jurisprudence, by A. B. Arnold, M.D.; pathology of inebriety, by Joseph Parrish, M.D.; hypodermic ergot in post-partum hemorrhage, by P. C. Williams, M.D.; significance of the præ-systolic murmur, by Frank Donaldson, M.D.; leukæmia, or leucocythæmia, by Henry R. Noel, M.D.; case of multiple fracture, by L. McLane Tiffany, M.D.; reform in medical education, by C. W. Chancellor, M.D.; little people as aids to diagnosis and treatment, by J. R. Uhler, M.D.; small-pox, by J. Summerfield Conrad, M.D., (illustrated;) the connection between excessive nerve and brain worry and bodily disease, with pathology and treatment of cancers, tumors, etc., by J. J. Caldwell, M.D.

---

The Climate and Diseases of the Gulf-Coast of the Florida Peninsula, with Remarks on the Former in Relation to Pulmonary Tuberculosis. By J. P. Wall, M.D., Tampa, Florida. Pamphlet; pp. 16.

